# Electrosynthesis of polydopamine-ethanolamine films for the development of immunosensing interfaces

**DOI:** 10.1038/s41598-021-81816-1

**Published:** 2021-01-26

**Authors:** Luís C. Almeida, Tânia Frade, Rui D. Correia, Yu Niu, Gang Jin, Jorge P. Correia, Ana S. Viana

**Affiliations:** 1 Centro de Química Estrutural, Faculdade de Ciências da Universidade de Lisboa, 1749-016 Campo Grande, Lisbon, Portugal; 2 NML, Beijing Key Laboratory of Engineered Construction and Mechanobiology, Institute of Mechanics, Chinese Academy of Sciences, Beijing, 100190 China

**Keywords:** Electrochemistry, Surface chemistry, Bioconjugate chemistry, Sensors

## Abstract

We report a straightforward and reproducible electrochemical approach to develop polydopamine-ethanolamine (ePDA-ETA) films to be used as immunosensing interfaces. ETA is strongly attached to polydopamine films during the potentiodynamic electropolymerization of dopamine. The great advantage of the electrochemical methods is to generate the oxidized species (quinones), which can readily react with ETA amine groups present in solution, with the subsequent incorporation of this molecule in the polymer. The presence of ETA and its effect on the electrosynthesis of polydopamine was accessed by cyclic voltammetry, ellipsometry, atomic force microscopy, FTIR and X-ray photoelectron spectroscopy. The adhesive and biocompatible films enable a facile protein linkage, are resilient to flow assays, and display intrinsic anti-fouling properties to block non-specific protein interactions, as monitored by real-time surface plasmon resonance, and confirmed by ellipsometry. Immunoglobulin G (IgG) and Anti-IgG were used in this work as model proteins for the affinity sensor. By using the one-step methodology (ePDA-ETA), the lower amount of immobilized biorecognition element, IgG, compared to that deposited on ePDA or on ETA post-modified film (ePDA/ETA), allied to the presence of ETA, improved the antibody-antigen affinity interaction. The great potential of the developed platform is its versatility to be used with any target biorecognition molecules, allowing both optical and electrochemical detection.

## Introduction

Immunosensors, based on the affinity reactions between an immobilized antibody and a target antigen, are extremely powerful and sensitive tools for disease control, environmental screening, and food quality monitoring^1^. There are, however, major challenges that must be overcome to achieve an efficient transduction interface in biosensors. These include a proper linkage of the biorecognition element to the surface, biocompatibility to avoid protein denaturation upon adsorption, and ability to prevent non-specific interactions with interferent proteins, during affinity assays^2^.

Optical biosensors often require the use of gold surfaces, which besides their unique optical properties (e.g. surface plasmon resonance phenomenon), can be efficiently and reproducibly modified with target biomolecules. There are several chemical and electrochemical functionalization approaches commonly used to attach biomolecules, such as the spontaneous adsorption of self-assembled monolayers (SAMs) of sulfur derivatives^3–5^, and the electrodeposition of conducting polymers^6,7^. Among the conducting polymers, poly(pyrrole), poly(aniline) and poly(ethylenedioxythiophene) are some of the most investigated ones, concerning biosensors with electrochemical transduction, due to their renowned electrosynthesis and conducting properties. In particular, poly(pyrrole) has been extensively used as a stable and conductive entrapment matrix^8^. However, in order to covalently couple biomolecules to this polymeric matrices, further steps must be followed, namely, previous functionalization of monomer or modification of the electrodeposited polymer (e.g. addition of COOH group). Then, chemical activation of the suitable groups in the polymer is frequently carried out, using the standard carbodiimide hydrochloride (EDC) / N-hydroxysulfosuccinimide (NHS) method^6^, to covalently link the biomolecules. This very same activation method is also frequently employed for the covalent attachment of biomolecules onto SAMs^4^. The great advantage of using conducting polymers regarding SAMs is the larger number of binding sites^9^, however, functionalized polymer matrices may not be as conducting and reproducible as desired, so the search for other polymers bearing active sites for biomolecule attachment has increased in the last years. A good example is polydopamine (PDA)^10–12^, a bioinspired polymer, with recognized excellent adhesion properties. Indeed, the chemical linkage of amine groups to the quinone moieties of PDA, through Michael addition or base Schiff reaction is widely reported in the literature^13^, supporting the versatility of these universal coatings. Lee et al.^14^ have thoroughly discussed the covalent conjugation between PDA quinone groups and nucleophiles (such as trypsin), demonstrating that it is a function of the number of catechol/quinone species, at a given pH, and of the pKa of the target nucleophile. Moreover, they proved that amine linkage to quinone moieties is more resistant to hydrolysis, than other popular functionalization strategies, including the NHS–ester approach. Despite of the large number of published works on PDA based films, its chemical composition is not precisely known. It is widely acknowledged that oxygen-driven polymerization of dopamine yields disorganized, poorly conductive and chemically heterogeneous films^11^. Catechol and quinone functional groups coexist, as well as different N-substituted rings^11,15, 16^. Recent studies, revealed that electrosynthesis of polydopamine yield more homogeneous films with better electrochemical properties suitable for electrochemical biosensing interfaces than the standard chemical synthesis, highlighting the great potential of electrochemical methods to better tune the chemical and physical properties of polydopamine^15,17^. In addition, it was also demonstrated by combining the electropolymerization processes with gravimetry^18^, that PDA has a much higher affinity for immunoglobulin G (IgG) than poly(pyrrole), due to the presence of the adhesive functionalities.

While the recognized adhesion properties of PDA films are a major advantage regarding the robust linkage of proteins, it can be a drawback when preparing affinity sensors, since the remaining active sites in the polymer, after bioreceptor immobilization, will be available for non-specific interactions with interferents. Due to the intrinsic reactivity of dopamine, there is the possibility of preparing “One-Pot” functional coatings, either chemically^11^, or electrochemically^19,20^, containing target molecules or biomolecules. The control over wettability, for instance, has been successfully achieved by the creation of superhydrophilic/superhydrophobic antifouling coatings, through the addition of specific compounds (e.g. sodium periodate or copper sulfate^21^ or diamines^22^, hyaluronic acid^23^) during dopamine or catechol polymerization.

**Figure 1:**
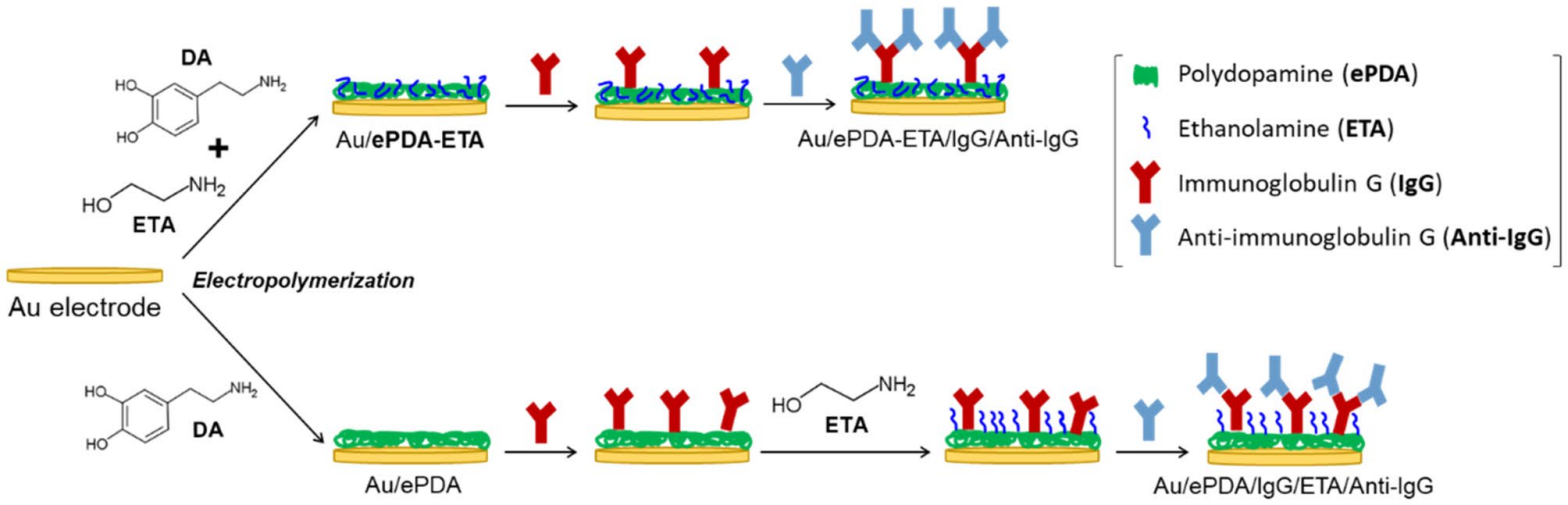
Schematic illustration of the gold modification steps for the construction of the polydopamine-ethanolamine immunosensing interface, using one-step or post-modification strategies.

The goal of this work is to provide a facile and very reproducible method to create a uniform polymeric interface with active sites for biomolecule covalent attachment, also with the ability to inhibit non-specific interactions, during protein affinity reactions. For that purpose, the potentiodynamic synthesis of polydopamine is carried out in the presence of ethanolamine (ETA). This small molecule has been used to block the remaining activated surface binding groups (e.g. carbodiimide activated COOH groups in SAMs^24,25^), which implies a multi-step approach. The immobilization of ethanolamine through its amine groups, enriches the surface with hydroxyl functionalities, known to prevent the protein non-specific adsorption in affinity reactions. Hereby, by applying a potential to the gold electrode we have a better control over the polymerization of dopamine (ePDA) as well as on the available quinones over the surface, that can readily react with the amine group of ETA in solution, to prepare the functional polymer (ePDA-ETA) suitable for affinity sensors. Post-modification of ePDA film with ETA was also carried out for comparison. The overall procedure to build-up the immunosensing interfaces is schematically presented in Fig. 1. Immunoglobulin G and Anti-immunoglobulin G were used as model proteins to evaluate the efficacy of this universal interface, that can be adapted to any trial of interest.

## Results and discussion

### Electropolymerization of dopamine in the presence of ethanolamine

Polydopamine films were potentiodynamically synthesized using a fast scan rate (200 mV s^–1^) and a low number of potential cycles (6 cycles)^15^ to obtain a thin, uniform and reproducible polymeric layer that is suited for post-modification with proteins and appropriate for electrochemical and optical detection of affinity biosensor recognition events. To ensure an electrochemical-driven polymerization, the electrolyte solutions were deaerated, avoiding any oxygen-driven polymerization of dopamine that may result in more heterogeneous and less organized films.

**Figure 2:**
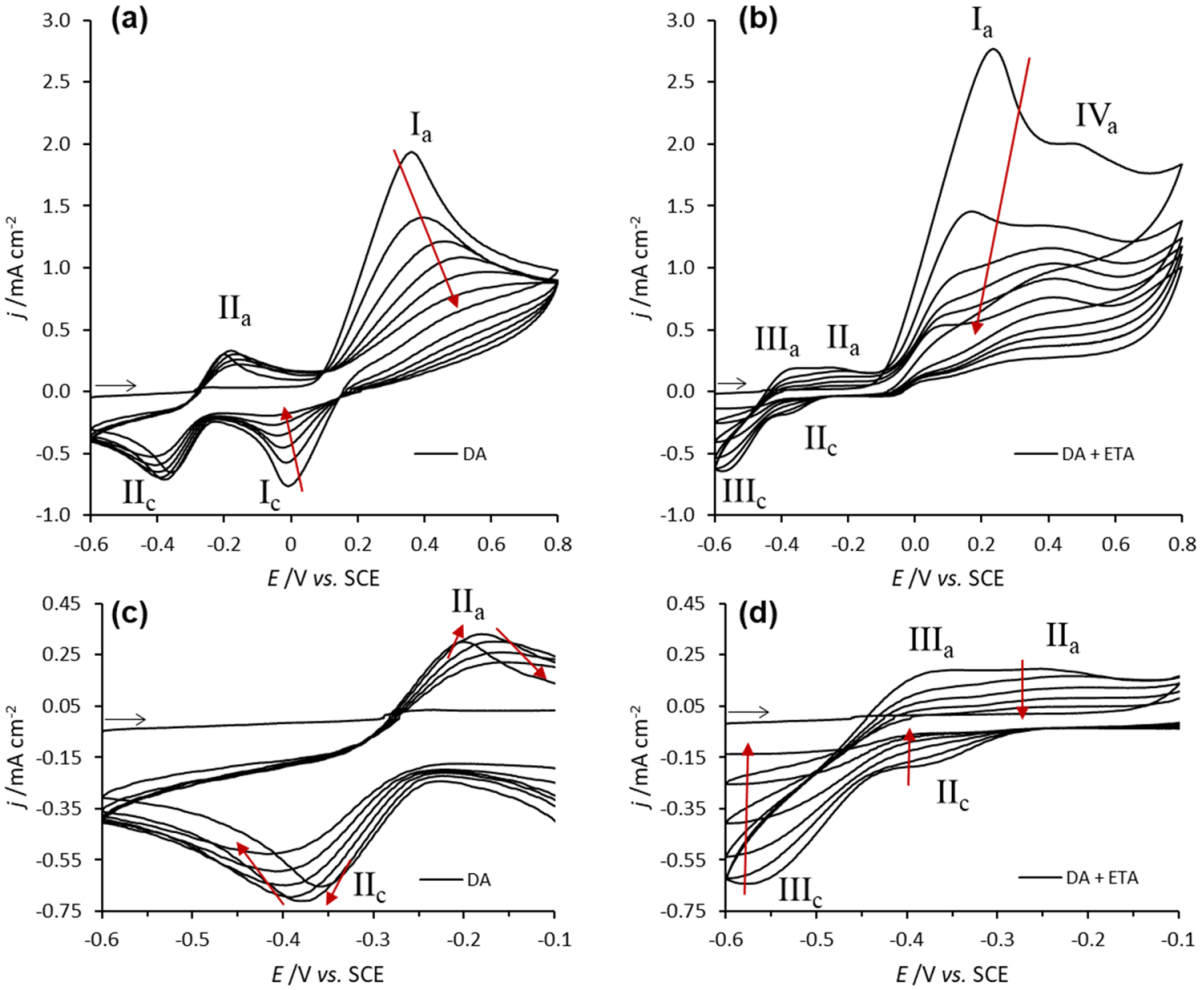
Potentiodynamic polymerization of 5 mM dopamine in the absence (**a**) and presence (**b**) of 100 mM ethanolamine, carried out at 200 mV s^−1^ for 6 potential cycles in deoxygenated CPB. Cyclic voltammograms (**c**) and (**d**) are amplifications of (**a**) and (**b**) respectively.

**Figure 3:**
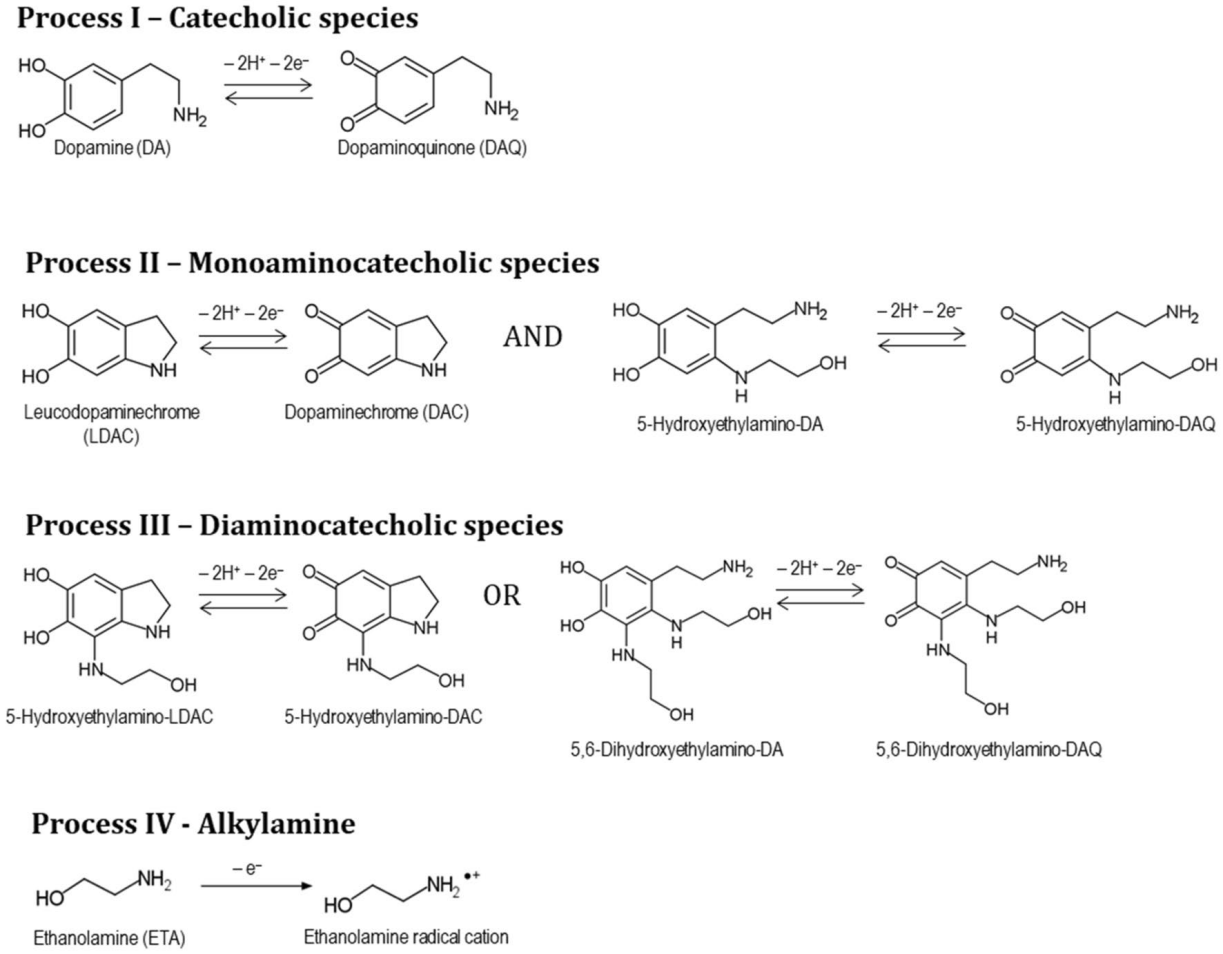
Electrochemical processes of catecholic (I), monoaminocatecholic (II), diaminocatecholic (III), and alkylamine (IV) species possibly occurring in the electropolymerization of dopamine in the presence of ethanolamine.

The incorporation of ethanolamine into polydopamine matrices, as mentioned, is envisaged as a strategy to inhibit the non-specific adsorption that occurs at the adhesive polydopamine surface, upon the immobilization of the biorecognition protein. To achieve such purpose, the electropolymerization of dopamine was performed in the presence of several concentrations of ethanolamine (10, 50 and 100 mM). Figure 2 shows the cyclic voltammograms of dopamine electropolymerization in the absence of ethanolamine, and in the presence of the highest concentration of ethanolamine studied (100 mM), where the effect of this additive can be clearly depicted. As previously reported^15^, two redox processes attributed to quinone/hydroquinone (Q/HQ) species in solution can be observed during dopamine electropolymerization in the absence of ethanolamine (Fig. 2a,c). Redox process I, occurring at E_1/2_ = 0.20 V, corresponds to the oxidation of dopamine to dopaminoquinone, and its respective reduction, as represented in the scheme of Fig. 3. These redox peaks decrease upon cycling which proves the formation of a low conducting polydopamine layer (Fig. 2a). As seen by the first anodic scan, process II is not present at the beginning of the electropolymerization. It is known that only after electrogeneration of dopaminoquinone, this reactive quinone undergoes a chemical step (intramolecular cyclization) originating an indoline bicyclic species, leucodopaminochrome (Fig. 3), that was not present in the initial monomer solution. Due to the electrodonating effect of the nitrogen atom attached to the catechol ring in the position 5, the oxidation of leucodopaminochrome (II_a_) and the reduction of its corresponding redox pair, dopaminochrome (II_c_), occur at significantly lower potential values than the process I. These two indoline-type species accumulate in solution during polymerization, which justifies the increase in peak currents II_a_ and II_c_ in the first three potential cycles (Fig. 2c). From the third to the last potential cycle, the intensity of process II decreases due to polymer thickening that hinders charge transfer across the film.

The presence of ethanolamine in the electrolyte solution causes a drastic effect on the original cyclic voltammograms of dopamine electropolymerization, as depicted in Fig. 2b,d. The previously identified Q/HQ redox processes I and II are shifted towards lower potential values due to the increase of pH, caused by the basic behavior of ethanolamine in solution (pH  10.2). In the presence of this compound, process I_a_ occurs at E_pa_ = 0.23 V (1^st^ potential cycle), whereas process II occurs at E_1/2_ =  − 0.29 V. In fact, an identical shift is observed when dopamine is electropolymerized in alkaline media (Figure S1, Supplementary material), where redox process I occurs at E_1/2_ = 0.23 V and process II at E_1/2_ =  − 0.33 V. Besides the potential shifts in the Q/HQ conversions, the process I became completely irreversible in the presence of ethanolamine (Fig. 2b), which indicates a very fast consumption of the electrogenerated dopaminoquinone into aminocatecholic species (N-substituted catecholic species). This is expected in alkaline medium since the deprotonation of amines and their subsequent nucleophilic attack to the quinone molecules is more favorable compared to the neutral medium^14,26^. This is true for the nucleophilic reaction of ethanolamine to the catechol ring, as well as to the intramolecular cyclization of dopamine. The nucleophilic attack of ethanolamine may occur through Michael addition (position 5 of catechol ring) or Schiff-base reaction to the carbonyl groups (position 1 or 2)^13^. In the latter case, an iminoquinone is formed and its redox potential is expected to be overlapped with Process I^27^, therefore difficult to distinguish by cyclic voltammetry. In contrast, the redox processes of aminocatecholic species during electropolymerization are ascribed to processes II and III (Fig. 3). Redox process II occurring at E_1/2_ =  − 0.33 V, is attributed to monoaminocatechols, namely, the indoline-type species originated from intramolecular cyclization of dopamine and also to the hydroxyethylamino-dopamine species, arising from ethanolamine nucleophilic attack at the position 5 of the catechol ring^28^. Redox process III, occurring at E_1/2_ =  − 0.47 V, is assigned to Q/HQ species that must have been further attacked by ethanolamine in the available positions of the quinone ring, which justifies the low redox potential value. Figure 3 shows possible structures of diaminocatechols, namely, the adduct of DAC with ethanolamine and the adduct of dopamine with two ethanolamine molecules. In fact, the new redox process III is not observed when dopamine is electropolymerized in alkaline medium in the absence of ethanolamine (Figure S1, Supplementary material). Only when using a high concentration of ethanolamine (100 mM), multiple attacks to the quinone rings should become more frequent and thus observable during electrochemical growth. The formation of aminophenolic adducts reflects an effective covalent bond between ethanolamine and the monomeric species that contribute to the building of the hybrid polymer. Besides, an irreversible oxidation process, at *ca.* 0.5 V (IV_a_), is depicted during the electropolymerization process only in the presence of ethanolamine (Fig. 2b). A control assay was performed only in the presence of 100 mM ethanolamine (in the absence of dopamine) (Figure S2, Supplementary material), confirming the presence of process IV_a_. This was assigned to the irreversible oxidation of ethanolamine to its radical cation^29,30^. Due to its reactive nature, it is expected that this radical may participate in the formation of hybrid oligomers of dopamine and ethanolamine, resulting in new ePDA-ETA polymers. Therefore, these polymers should contain additional alkyl hydroxyl groups with anti-fouling properties, required to avoid the non-specific adsorption of interferent proteins.

**Figure 4:**
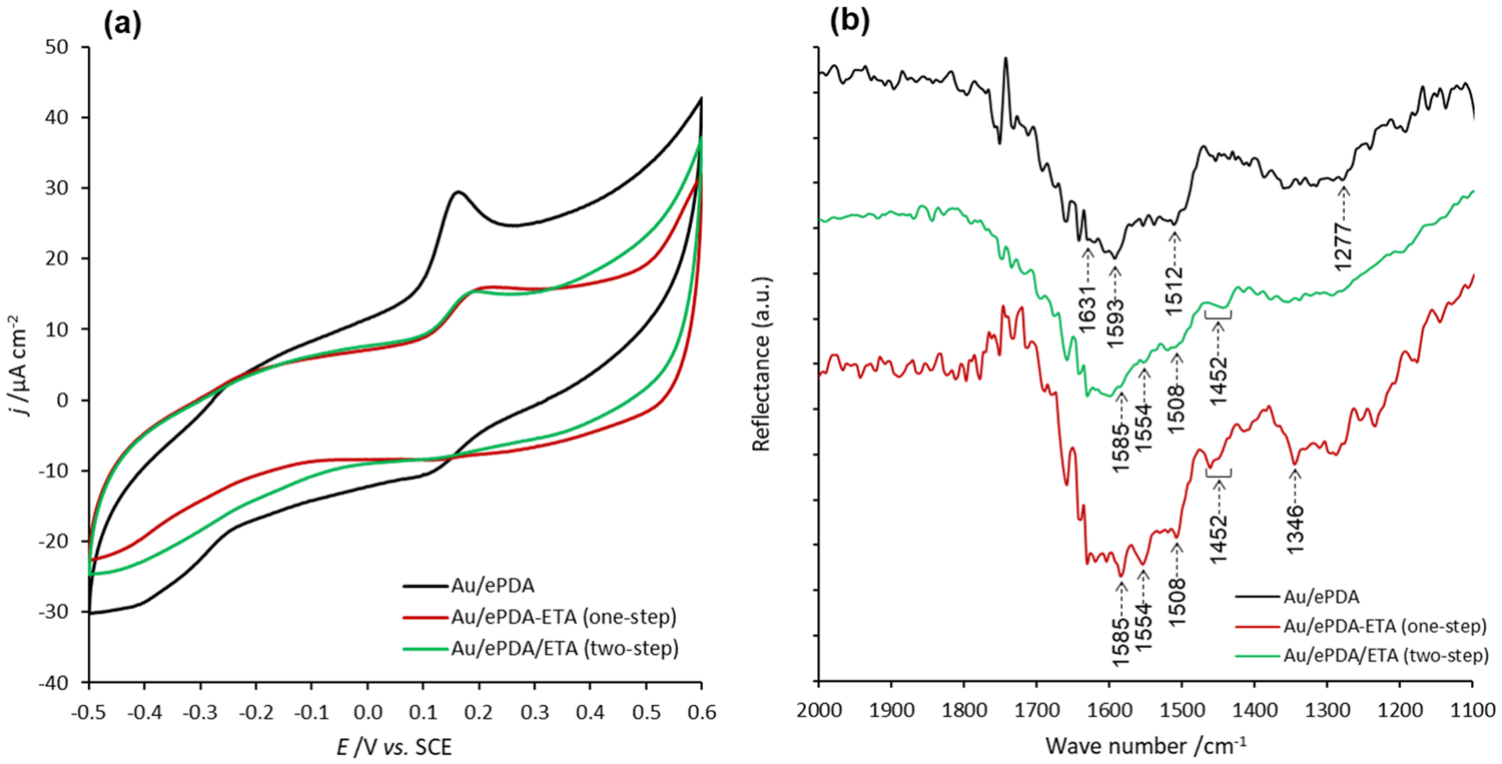
Cyclic voltammograms (**a**) and FTIR spectra (**b**) of gold electrodes modified with polydopamine (Au/ePDA), poly(dopamine-ethanolamine) (Au/ePDA-ETA) and polydopamine film after incubation in ethanolamine solution (Au/ePDA/ETA). Cyclic voltammograms were recorded at 50 mV s^−1^ in CPB pH 7.0.

The formation of electroactive polymers on the surface of the gold was confirmed by cyclic voltammetry, performed in monomer-free electrolyte solution (Fig. 4a). The pristine ePDA film cyclic voltammogram displays a well-defined redox process at E_1/2_ = 0.14 V attributed to Q/HQ conversions of the polymeric chains of dopamine, as reported previously^15,31^. However, as mentioned above this process may also account for iminoquinones/aminophenols redox conversion, as a result of Schiff base reactions^27^. This main redox process is found to be greatly affected when ethanolamine is added to the electrosynthesis solution (one-step, ePDA-ETA) or when the electrosynthesized polymer is incubated in an ethanolamine solution (two-step, ePDA/ETA), as it can be depicted by the current decrease of such process in both cyclic voltammograms of ePDA-ETA and ePDA/ETA (Fig. 4a). A lower electroactivity of the ethanolamine modified polymers strongly suggests that the nucleophilic attack of ethanolamine to the polydopamine affects the Q/HQ redox process.

Pristine and ethanolamine-modified films were characterized by FTIR (Fig. 4b), confirming the incorporation of ethanolamine in ePDA using either a one-step or a post-modification approach. ePDA spectra displays bands at 1631, 1593, 1512 and 1277 cm^−1^, previously reported^15^ for this polymer. The two most intense peaks, at 1631 and 1593 cm^−1^, correspond to aromatic asymmetric C = C stretching modes of the dihydroxyphenol ring, while less intense aromatic symmetric C=C stretching (1512 cm^−1^) and phenolic C–OH stretching (1277 cm^−1^) modes can also be depicted. The band at 1593 cm^−1^ may also have the contribution of NH_2_ scissoring mode^32^. After incubation of ePDA film with ethanolamine, new features appear in the ePDA/ETA spectra suggesting the presence of a small amount of ethanolamine on the polymer film. The modification is more evident when a one-step electrochemical approach is used, indicating the incorporation of a larger amount of ethanolamine during electropolymerization, that leads to better-defined peaks at frequencies typical of ethanolamine. The three characteristic IR peaks reported for free^33,34^ and surface-adsorbed^35,36^ ethanolamine, can be identified in both ethanolamine-modified films spectra (Fig. 4b), namely, the coupled deformation modes of NH_2_ with CNH (1585 cm^−1^), CH_2_ with NCH (1442–1462 cm^−1^) and OCH with CCH (1340–1346 cm^−1^). Moreover, the peaks appearing at 1554 and 1508 cm^−1^ indicate the presence of *p*-quinoid and *p*-benzenoid rings, respectively, similar to those reported for polyaniline^37^, poly(N-ethylaniline)^38^ and poly(o-aminophenol)^39^, which corroborates the covalent linkage of ethanolamine to the dihydroxyphenol rings of the pristine ePDA.

**Figure 5:**
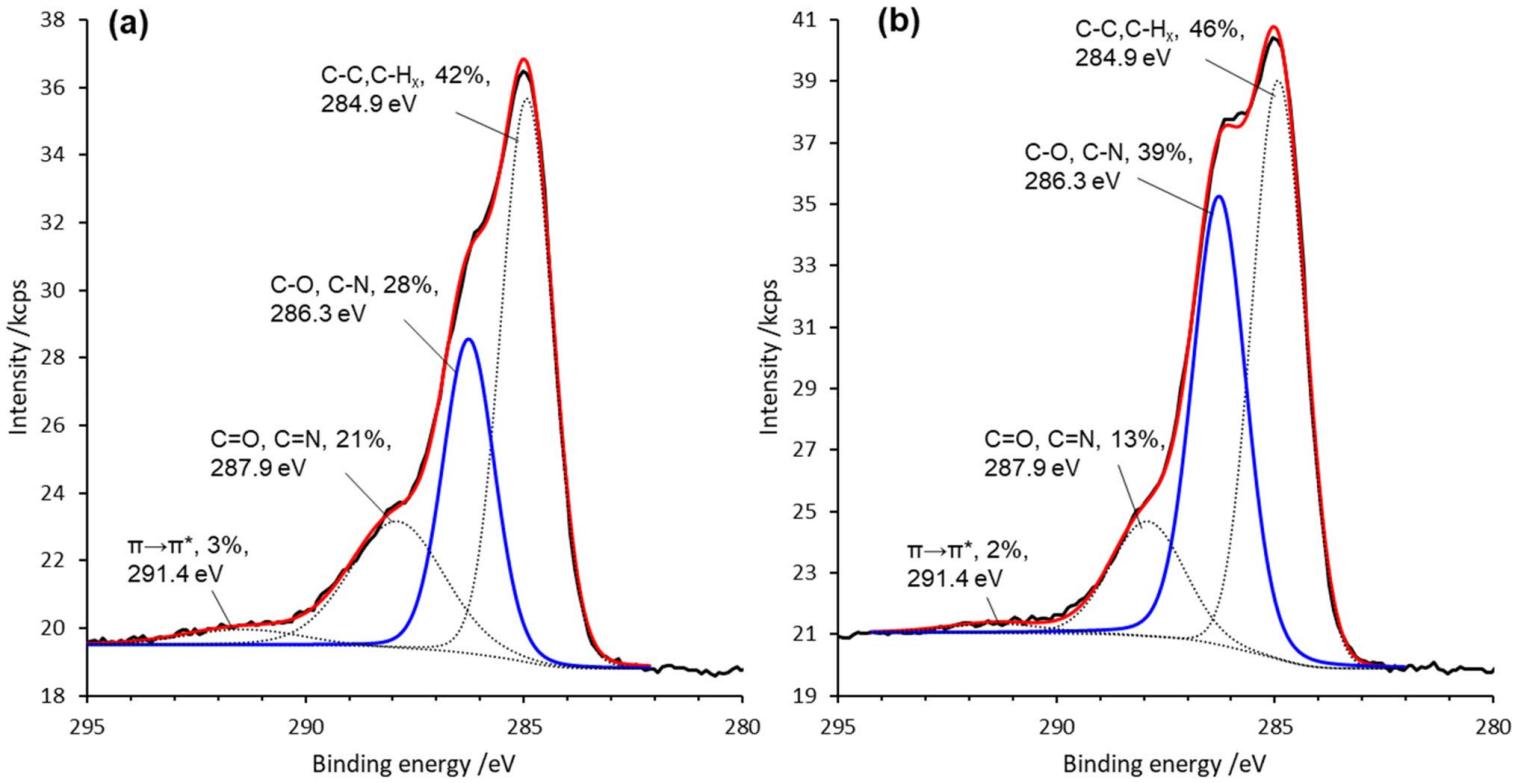
XPS spectra of C 1s region of pristine polydopamine (ePDA) (**a**) and one-step modified polydopamine (ePDA-ETA) (**b**).

X-ray photoelectron spectroscopy (XPS) analysis was carried out on ePDA and ePDA-ETA films aiming at characterizing the bonds established during the one-step modification and to further confirm the presence of ethanolamine in ePDA polymer. The atomic ratios N/C: 0.11 (ePDA) and 0.13 (ePDA-ETA), and O/C: 0.28 (ePDA) and 0.26 (ePDA-ETA), are identical for both films, and are in agreement within the expected values for polydopamine films^21,40^, since N/C and O/C atomic ratios for dopamine are 0.125 and 0.250, respectively. The main differences between the two polymers arise from C 1s spectral region shown in Fig. 5 (N 1 s and O 1 s spectral regions are provided on Supplementary material, Figure S3). The C 1 s spectra have been fitted into four main components attibuted to C–C, C-H_x_ (284.9 eV), C-O, C-N (286.3 eV), C=O, C=N (287.9 eV) and π → π* (291.4 eV)^40,41^. It is possible to observe a significant increase of the C-O, C-N component in ePDA-ETA spectrum (Fig. 5b) relatively to pristine ePDA spectrum (Fig. 5a). The (C-O, C-N)/(C–C, C-H_x_) ratios for ePDA-ETA and ePDA are 0.85 and 0.67, respectively, accounting for a higher number of C-N and C–OH bonds, arising from the successful attachment of aliphatic ethanolamine molecules.

### IgG adsorption on ePDA and ethanolamine-modified films

**Figure 6:**
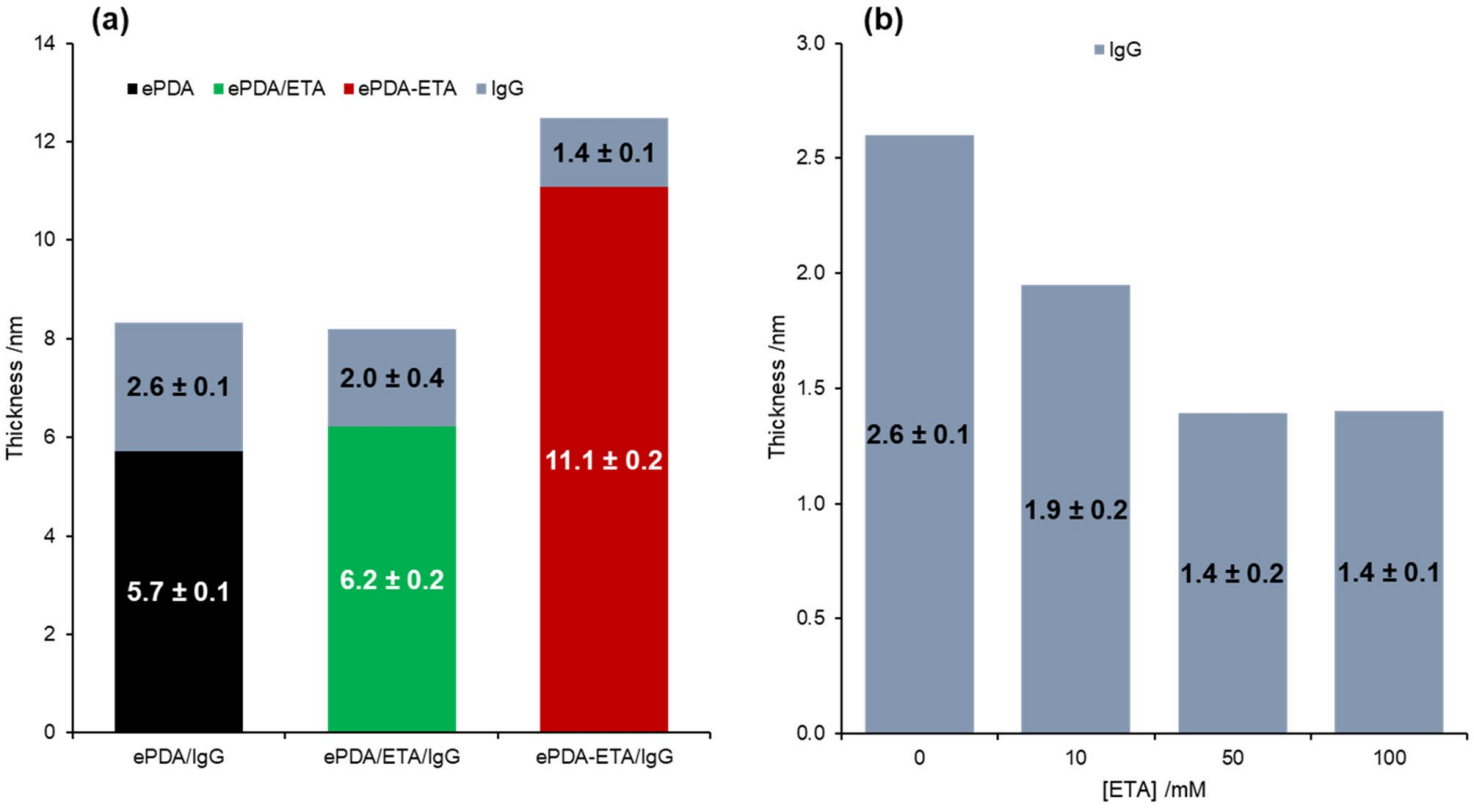
(**a**) Optical thicknesses of pristine polydopamine (ePDA), two-step modified polydopamine (ePDA/ETA) and one-step modified polydopamine (ePDA-ETA), before and after IgG incubation on the several polymers. (**b**) Effect of ethanolamine concentration during ePDA-ETA electrosynthesis on the IgG layer thickness.

**Figure 7:**
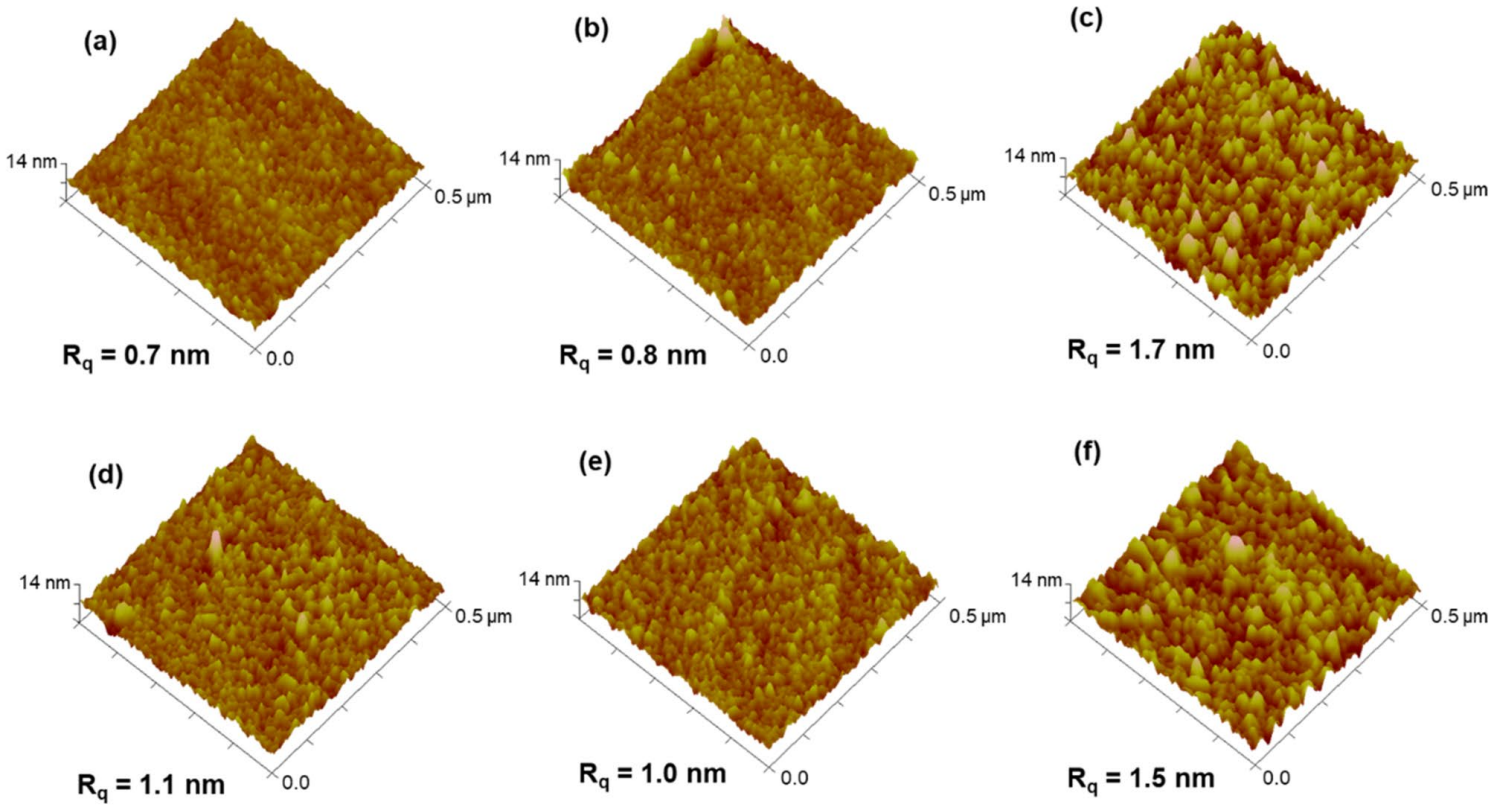
AFM 3D images of modified gold with ePDA (**a**), ePDA/ETA (**b**), ePDA-ETA (**c**), ePDA/IgG (**d**), ePDA/ETA/IgG (**e**) and ePDA-ETA/IgG (**f**).

The polymers were incubated in IgG solution and analyzed by ellipsometry (Fig. 6) and AFM (Fig. 7), to evaluate the optical thickness increase and morphological changes occurring at the ePDA interface upon the adsorption of this protein.

The estimated thickness for ePDA film, *ca.* 5.7 nm (Fig. 6a), confirms the formation of a thin polymeric coating on gold, in agreement with other reports of electrochemically synthesized polydopamine^15^ on carbon electrodes. The AFM morphological image of ePDA (Fig. 7a) shows a granular and uniform polymer with a slightly higher root-mean-square roughness (R_q_) than that of bare gold (R_q_ = 0.5 nm^42^). After incubation with ethanolamine, there is a very small, yet consistent, thickness increase relative to the ePDA layer (Fig. 6a), which corroborates the modification of the polymer surface with this molecule using the two-step strategy (ePDA/ETA). As suggested by FTIR data, only a small amount of ethanolamine is foreseen to be present at ePDA/ETA modified film, and indeed the roughness of this interface is very similar to the ePDA film before modification (Fig. 7). Even so, it is possible to observe a less uniform surface of ePDA/ETA (Fig. 7b), when compared to ePDA film topography (Fig. 7a). The electrochemically synthesized film by the one-step strategy (ePDA-ETA) is considerably thicker (11.1 nm, Fig. 6a) in comparison with ePDA, due to the faster electropolymerization growth in a more alkaline media provided by ethanolamine, as discussed above. Consequently, ePDA-ETA (Fig. 7c) is considerably rougher than ePDA or ePDA/ETA.

Polydopamine films were incubated in IgG solution and thoroughly washed to remove the protein molecules that did not robustly attach at pristine and ethanolamine-modified polymeric surfaces. It is expected that IgG molecules, which contain amine groups, may also establish covalent bonds with oxidized catechol moieties in the polymer, similarly to ethanolamine. Due to the complexity and richness of protein surface functionalities, it is also conceivable that intermolecular interactions may also contribute to the robust protein immobilization^18^. One can depict a small increase in the number of large features on ePDA/IgG (Fig. 7d) and ePDA/ETA/IgG (Fig. 7e), when compared to the same surfaces before IgG incubation, with a consequence increase in roughness. These small changes reflect the presence of well-distributed IgG over polymers following the granular film morphology. The similarity between the size of polymer grains and proteins, imaged by AFM, precludes the clear visualization of individual protein molecules. Nevertheless, the increase in thickness upon IgG incubation on ePDA and ePDA/ETA, retrieved by ellipsometry (Fig. 6a), confirms the great ability of the film to immobilize proteins, without the need of any chemical activation step. The thickness variation is higher for ePDA/IgG (2.6 nm) than for ePDA/ETA/IgG (2.0 nm) interface, proving that the two-step strategy diminishes the number of available binding sites for IgG, still allowing a significant protein coverage that is required for the following biorecognition event. We have also observed that ePDA films incubated in 5 or 100 mM ethanolamine solutions (Figure S4, Supplementary material) yield very similar protein thicknesses. Thus, the lowest ethanolamine concentration was used for the biorecognition reactions using the two-step ePDA/ETA interfaces.

In the case of the ePDA-ETA interface, the surface morphology was kept after IgG adsorption, with a slight decrease of R_q_ values (Fig. 7f). In fact, the increase in thickness for the protein layer on ePDA-ETA is only 1.4 nm, which is inferior than the previous polymer/IgG interfaces. This lower IgG coverage indicates that the amount of incorporated ethanolamine, using the one-step strategy, is more effective in reducing the number of sites for IgG adsorption in the polymer surface, certainly providing a better distribution and more spaced molecules at the biorecognition interface^4^. Besides, as shown in Fig. 6b, the concentration of ethanolamine was changed from 10 to 100 mM during the potentiodynamic polymerization of dopamine, clearly affecting the inhibition of protein adsorption on ePDA/ETA films. These observations demonstrate the suitability of the one-step electrochemical process for polymer functionalization, that promotes the nucleophilic attack of ethanolamine molecules to the generated quinones during the potential application.

### Evaluation of the immunosensor performance

**Figure 8:**
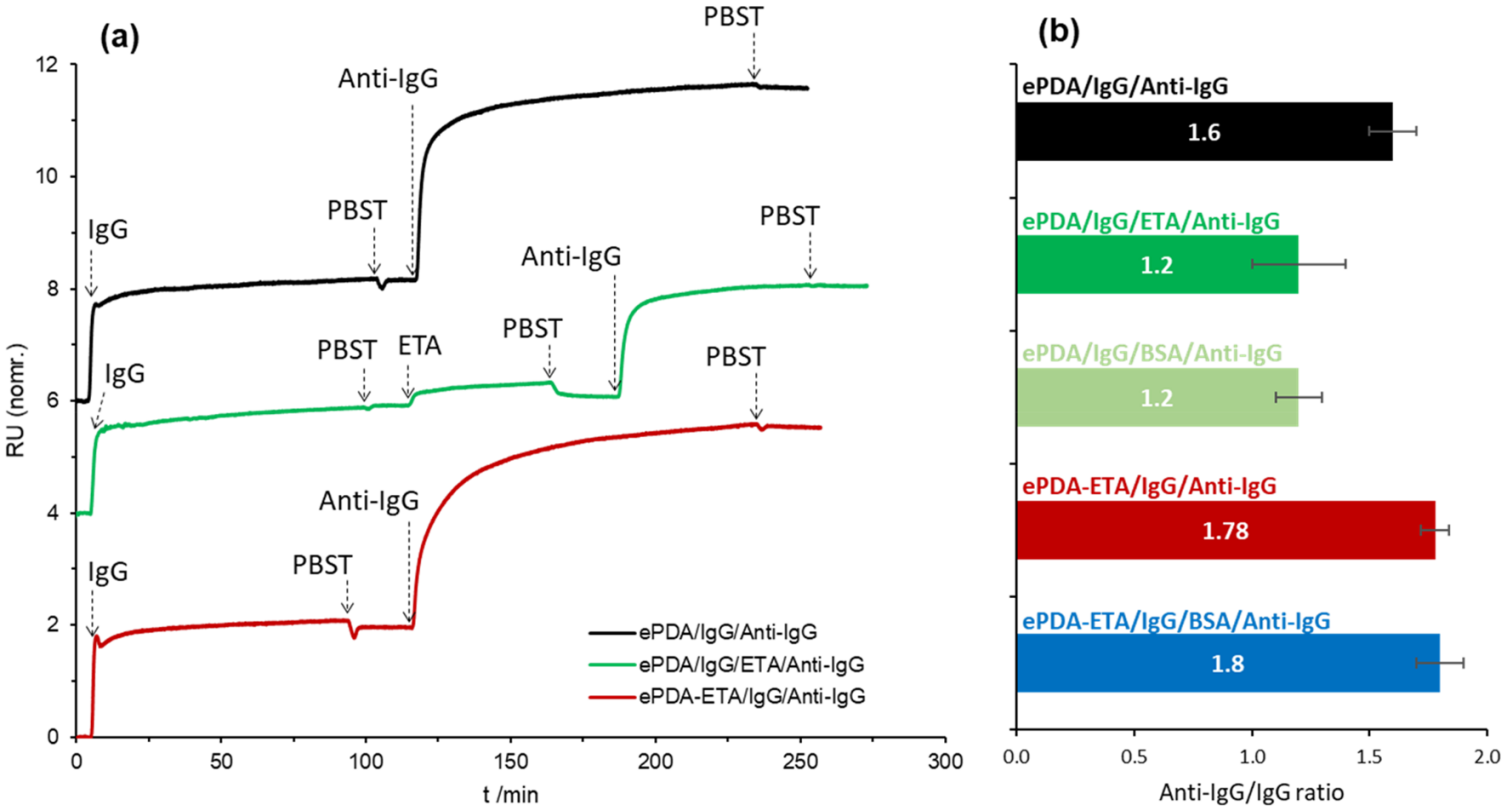
Representative real-time SPR curves showing the IgG and Anti-IgG interaction on gold surfaces modified with ePDA, in the absence and presence of a blocking step using either ETA or BSA; and with ePDA-ETA film, in the absence and presence of a blocking step using BSA (**a**); Anti-IgG/IgG ratios obtained from three independent SPR assays (**b**).

**Figure 9:**
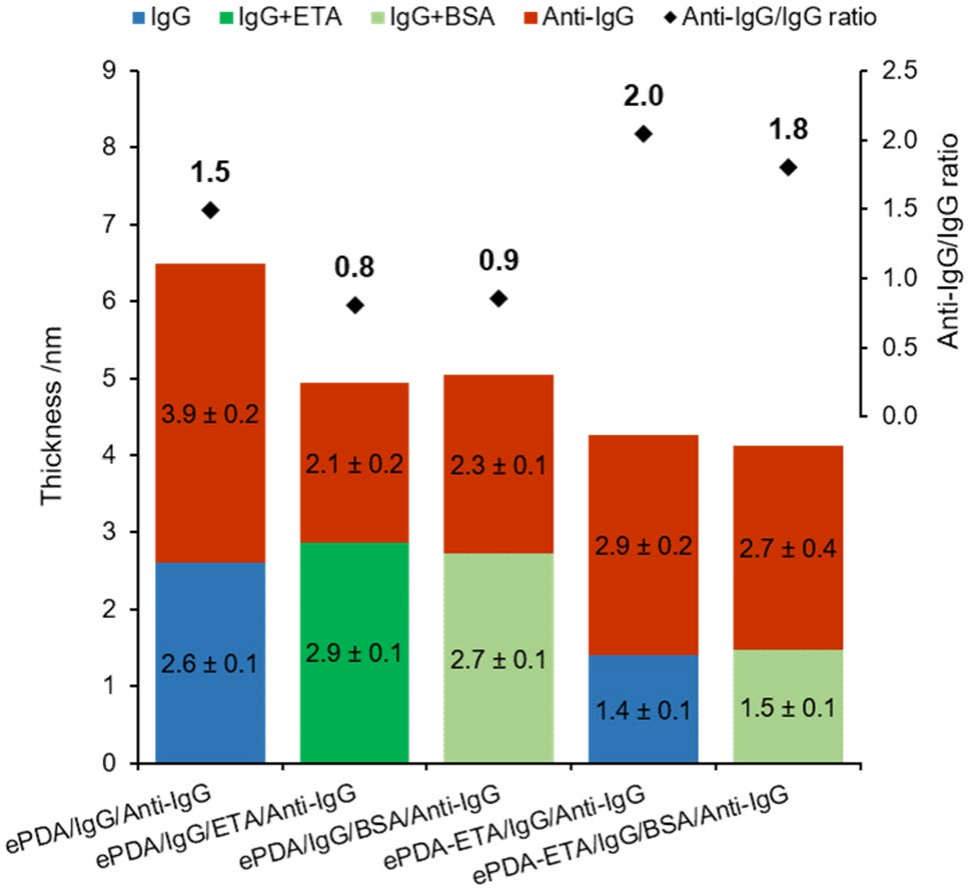
Optical thicknesses of IgG and Anti-IgG interaction on gold surfaces modified with ePDA, in the absence and presence of a blocking step using either ETA and BSA; and with ePDA-ETA film, in the absence and presence of a blocking step using BSA. Anti-IgG/IgG ratios, obtained from three independent ellipsometric assays, are also presented.

After evaluating the effect of ethanolamine on the interaction of proteins with the surface of the modified polydopamine films, these interfaces were tested in real-time SPR assays to access their performance as immunosensors (Fig. 8). As shown previously by the *ex-situ* ellipsometric results (Fig. 6a), the pristine polydopamine layer is able to capture IgG and preserve a stable bond even after extensive rinsing with buffer. This is also clear in the SPR curves (Fig. 8), since the decrease of optical signal is minimal after rising the surface that was exposed to IgG. This is crucial to ensure the lowest leaking of the capture antibody during the following steps of modification or detection. After interacting with Anti-IgG, the ePDA/IgG interface yields an Anti-IgG/IgG ratio of 1.6 (Fig. 8b), most probably due to the contribution of specific and non-specific protein interactions, the latter arising from the adhesive properties of the polydopamine films. Hence, upon the immobilization of IgG there might still be some polymer binding sites that can interact with Anti-IgG, accounting for a number of protein non-specific linkages. The *ex-situ* ellipsometric measurements, presented in Fig. 9, indicate that for the pristine ePDA film, the Anti-IgG layer is *ca.* 1.5 times thicker than the IgG layer, corroborating the real-time SPR data.

When a blocking step with ethanolamine is used, after IgG immobilization on ePDA (Fig. 8a, middle curve), there is no visible removal of IgG. Indeed, as expected for a small molecule, the SPR signal does not change significantly after the introduction of ethanolamine, as it is also corroborated by the estimated optical thicknesses for IgG and IgG/ETA layers, 2.6 and 2.9 nm, respectively (Fig. 9). The blocking of the polymer binding sites with ethanolamine can be inferred by the much lower variation of the optical signal caused by the capture of Anti-IgG, which causes a decrease of Anti-IgG/IgG ratio from 1.6 to 1.2 (Fig. 8). Thus, the Anti-IgG that is captured in this case must be specifically bonded to IgG. To confirm this result, a common blocking agent, BSA^5^, was also used to block the polymer binding sites that remain after IgG incubation. The same low ratio of 1.2 is observed (Fig. 8b), proving that ethanolamine can effectively replace protein-based blocking buffers.

The performance of the one-step synthesized ePDA-ETA film was also investigated as a biosensor interface, displaying an Anti-IgG/IgG ratio of 1.8 (Fig. 8b), which is considerably higher than the ratio obtained when using a pristine ePDA film in the absence or presence of a blocking step. This is a great improvement revealing that the incorporation of ethanolamine during the electrosynthesis improves the IgG spacing, allowing a better accommodation of the bonded Anti-IgG. Ellipsometric data agrees with this result, showing an Anti-IgG layer two times thicker than the IgG layer (Fig. 9). More importantly, this interaction is specific since the introduction of a blocking step with BSA did not affect (within the experimental error) the Anti-IgG/IgG ratio (Figs. 8b and 9). The electrosynthesis of a polydopamine film in the presence of a simple blocking agent is then an efficient strategy to be used in the reproducible preparation of ready-to-use platforms as immunosensing interfaces, avoiding multi-step procedures, commonly required for protein immobilization and for the detection of specific antigen–antibody interaction.

## Conclusions

Hereby, we present a successful one-step modification strategy that allows a fast and reproducible synthesis of a ready-to-use platform in immunosensors without the need for additional chemical coupling reactions, during biomolecule immobilization, or blocking steps throughout protein affinity interactions. Cyclic voltammograms, recorded during polydopamine electrosynthesis in the presence of ethanolamine, disclose new quinone/hydroquinone species, absent during the potentiodynamic growth of pure polydopamine. These redox reactions are assigned to species originated from the covalent coupling of ethanolamine to monomer or oligomers with the subsequent incorporation in the polymeric film. The modified polymers are less electroactive than the pristine ePDA film, supporting the reaction of surface quinones with amine groups of ethanolamine. FTIR proves the presence of ethanolamine in the polymeric matrix, which is more evident when performing the one-step electrochemical polymerization of dopamine with ethanolamine. XPS data corroborates the covalent attachment of ethanolamine to the polymers when using a one-step electropolymerization procedure.

A partial inhibition of IgG attachment to ePDA films modified with ethanolamine is achieved by one or two-step functionalization strategies, although the one-step electrochemical method proved to be the most efficient in incorporating ethanolamine into the polymer matrix, resulting in a decrease of protein surface coverage, as confirmed by ellipsometry. The slightly lower amount of protein avoids steric hindrance effects among biorecognition elements, increasing the number of specific antibody-antigen interaction. Real-time immunosensing assays by SPR show that incubation of ethanolamine, after protein immobilization, causes a similar blocking effect as standard BSA, and that ethanolamine incorporation during electrosynthesis greatly improve the detection ratio. Thus, the presence of ethanolamine does not disturb the direct attachment of antibody and promotes the specific recognition of its antigen, without the need of further blocking steps with protein-based buffers, during the biorecognition assay.

## Materials and methods

### Chemicals

Dopamine hydrochloride (DA, Sigma-Aldrich), ethanolamine (ETA, Fisher Scientific), immunoglobulin G from human serum (IgG, Sigma-Aldrich), polyclonal Anti-human immunoglobulin G (whole molecule) produced in goat (Anti-IgG, Sigma-Aldrich) and bovine serum albumin (BSA, Sigma-Aldrich) were all used without further purification. Citrate–phosphate buffer (CPB, pH 7.0) was prepared with 10 mM citric acid (Sigma-Aldrich) and 80 mM disodium hydrogen phosphate anhydrous (Merck). Phosphate buffer saline (PBS, pH 7.4, 0.01 M phosphate buffer containing 0.138 M sodium chloride and 0.0027 M potassium chloride), obtained from Sigma-Aldrich was used without or with 0.05% (v/v) of Tween20 (PBST). Ultrapure water, obtained from a purification MILLI-Q A10 system (18.2 MΩ cm), was used to prepare buffers and rinsing steps.

### Preparation of gold surfaces

In this work, two types of gold substrates were employed: SPR glass slides coated with polycrystalline gold (50 nm) purchased from Analytical µ-Systems, and borosilicate glass slides coated with polycrystalline gold (200 nm) manufactured by Arrandee. The implemented cleaning procedure is described elsewhere with slight modifications^2^. Before gold surface modifications, the SPR slides were rinsed with ultrapure water and ethanol, dried with nitrogen flux, and cleaned in a UV chamber for 40 min. For AFM, ellipsometry, and electrochemical experiments, the Arrandee gold slides were cleaned in a piranha solution (H_2_SO_4_/H_2_O_2_ = 3:1, v/v), washed with ultrapure water and ethanol, dried under nitrogen flux, flame-annealed under butane-oxygen torch, cleaned once more in piranha solution and finally rinsed with ultrapure water and dried again with nitrogen flux.

### Electrosynthesis of ePDA and ePDA-ETA films

The electropolymerization of dopamine was performed potentiodynamically, between − 0.6 V and + 0.8 V *vs.* SCE, for 6 potential cycles at a scan rate of 200 mV s^−1^, using a freshly prepared 5 mM dopamine solution in CPB buffer, deoxygenated for 30 min to avoid any oxygen-driven polymerization. For the one-step strategy, the electropolymerization of dopamine was carried out in the presence of ethanolamine (10, 50 and 100 mM). Whenever indicated in the text, pristine ePDA films were also incubated in 5 mM or 100 mM ethanolamine PBS solution for 3 h. The modified gold electrodes were washed with water and dried with nitrogen flux.

Electrochemical assays were performed in a three-electrode Teflon cell using a CHI600A Electrochemical Analyser potentiostat. A saturated calomel electrode (SCE) and a platinum foil served as the reference and counter electrodes, respectively. Geometric areas of the gold working electrodes are 0.58 cm^2^ for Arrandee slide and 3.1 cm^2^ for the SPR slide. All the electrolyte solutions were degassed with nitrogen before each experiment for 30 min.

### Surface plasmon resonance

SPR measurements were carried out in a BIOSUPLAR400T compact SPR equipment manufactured by Analytical μ-Systems coupled to a peristaltic pump from ISMATEC at a flow of 25 μL/min. The modified gold surfaces with ePDA or ePDA-ETA film were mounted onto the SPR equipment. Before the antigen–antibody assay, water and PBS were pumped into the cell to normalize the raw optical signals (resonance units, RU) according to the equation: RU_norm._ = RU/(RU_PBS_-RU_water_). Afterward, a 0.1 mg/mL IgG solution was pumped into the system and left to incubate till a stable optical signal was acquired. After PBST washing, a 0.1 mg/mL Anti-IgG solution was pumped and left to incubate again until the optical signal was stable. In some cases, a blocking step was also performed after IgG incubation, using 5 mM of ETA or 0.1 mg/mL of BSA. Data shown are the representative behavior of at least 3 independent experiments. The signal noise level was determined to be ≤ 1 RU.

### Ellipsometry

The optical parameters of the surfaces were measured by ellipsometry upon each modification step. Polymer surfaces were incubated with 0.1 mg/mL of IgG in PBS for 1.5 h, at room temperature, thoroughly rinsed with PBST and water, and dried under nitrogen flow, followed by immersion in 0.1 mg/mL of Anti-IgG in PBS for 1.5 h. An intermediate blocking step was performed for 30 min with 5 mM of ETA or 0.1 mg/mL of BSA, both in PBS, for some modified electrodes, as explained along the text.

Ellipsometric measurements were performed in air at an incidence angle of 70° using a SE 400 Ellipsometer (Sentech Intruments) equipped with a He–Ne laser (632.8 nm). The refractive index and extinction coefficient of the gold substrate was calculated assuming a semi-infinite two-phase model (gold/air). To estimate the refractive index, extinction coefficient and thickness of the polymeric films (with or without ETA), the experimental ellipsometric data was adjusted to a three-phase model (gold/film/air). The thicknesses of the protein layers were estimated by fixing its complex refractive index at 1.5 − 0i^5,43^ and adjusting the experimental ellipsometric data, assuming a multi-phase model (gold/film/protein/air).

### Atomic force microscopy

AFM measurements were performed in air at 23 ± 1 °C, using a Multimode 8 h microscope coupled to Nanoscope V (Digital Instruments, Bruker). The images were acquired in Peak Force Tapping (at a frequency of 2 kHz) using ScanAsyst mode, with Silicon Nitride ScanAsyst-Air probes (spring constant of *ca.* 0.4 N m^−1^, Bruker), at a scan rate of *ca.* 1.0 Hz.

### Fourier-transform infrared spectroscopy

FTIR spectra were obtained with a Nicolet Nexus 6700 FTIR spectrometer equipped with the Specular Apertured Grazing Angle module (SAGA). The SPR gold slides were used as substrates, selecting the sample mask with a 16 mm aperture. A number of 128 scans were collected for each sample using 4 cm^−1^ resolution.

### X-ray photoelectron spectroscopy

High resolution X-ray photoelectron spectra were acquired at TEMA, University of Aveiro, an Ultra High Vacuum (UHV) system with a base pressure of 2 × 10^−10^ mbar, fitted with a hemispherical electron energy analyzer (SPECS Phoibos 150), a delay-line detector and a monochromatic AlKα (1486.71 eV) X-ray source. The spectra were recorded at normal emission take-off angle and with a pass-energy of 20 eV, which provides an overall instrumental peak broadening of 0.5 eV. The effect of the electric charge was corrected with reference to the C 1s peak (285.0 eV)^44^. The spectra were deconvoluted with XPSPEAK software, using a Gaussian–Lorentzian peak shape and Shirley (or Linear) type background subtraction.

## Supplementary Information

Supplementary Information
