# Supplementary material for: Electrosynthesis of polydopamine-ethanolamine films for the development of immunosensing interfaces
Source: Sci Rep. 2021 Jan 26;11:2237. doi: 10.1038/s41598-021-81816-1 (PMC7838280; doi:10.1038/s41598-021-81816-1)
Supplement: Supplementary file 1 — Supplementary Information [file 41598_2021_81816_MOESM1_ESM.pdf]

Supplementary Material

Electrosynthesis of polydopamine-ethanolamine films for the development of immunosensing interfaces

Luís C. Almeida [1, ¥], Tânia Frade [1, ¥], Rui D. Correia [1, ¥], Yu Niu [2], Gang Jin [2], Jorge P. Correia [1], and Ana S. Viana [1,*]

[1] Centro de Química Estrutural, Faculdade de Ciências da Universidade de Lisboa, Campo Grande, 1749-016 Lisboa, Portugal;

[2] NML, Beijing Key Laboratory of Engineered Construction and Mechanobiology, Institute of Mechanics, Chinese Academy of Sciences, Beijing 100190, China;

¥ Authors contributed equally to this work

* Correspondence: anaviana@fc.ul.pt; Tel.: +351-217500000 (ext. 28437)

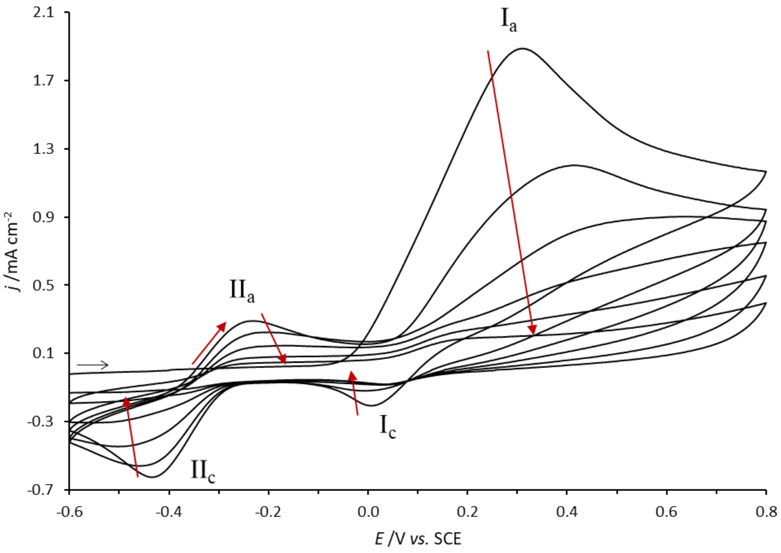

Fig. S1: Potentiodynamic polymerization of 5 mM dopamine, performed at 200 mV s^{-1} for 6 potential cycles in deoxygenated CPB (pH = 10.2).

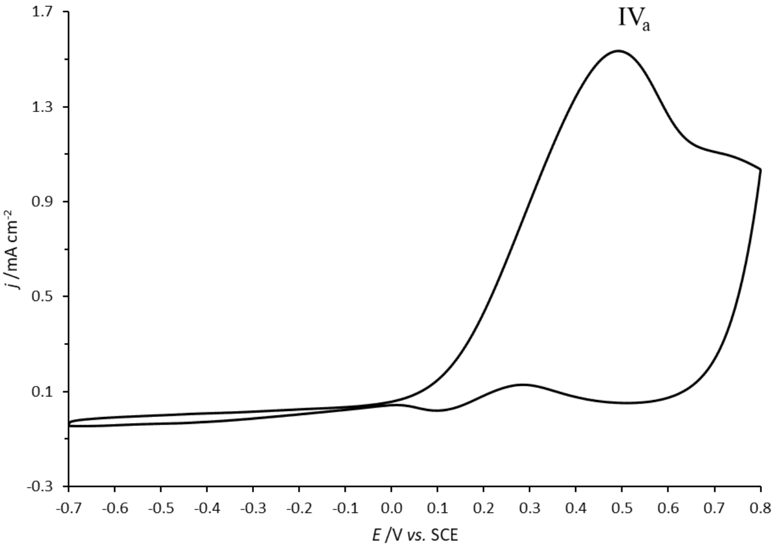

Fig. S2: Cyclic voltammetry of gold electrode, recorded at 200 mV s^{-1} in 100 mM ethanolamine solution (deoxygenated CPB).

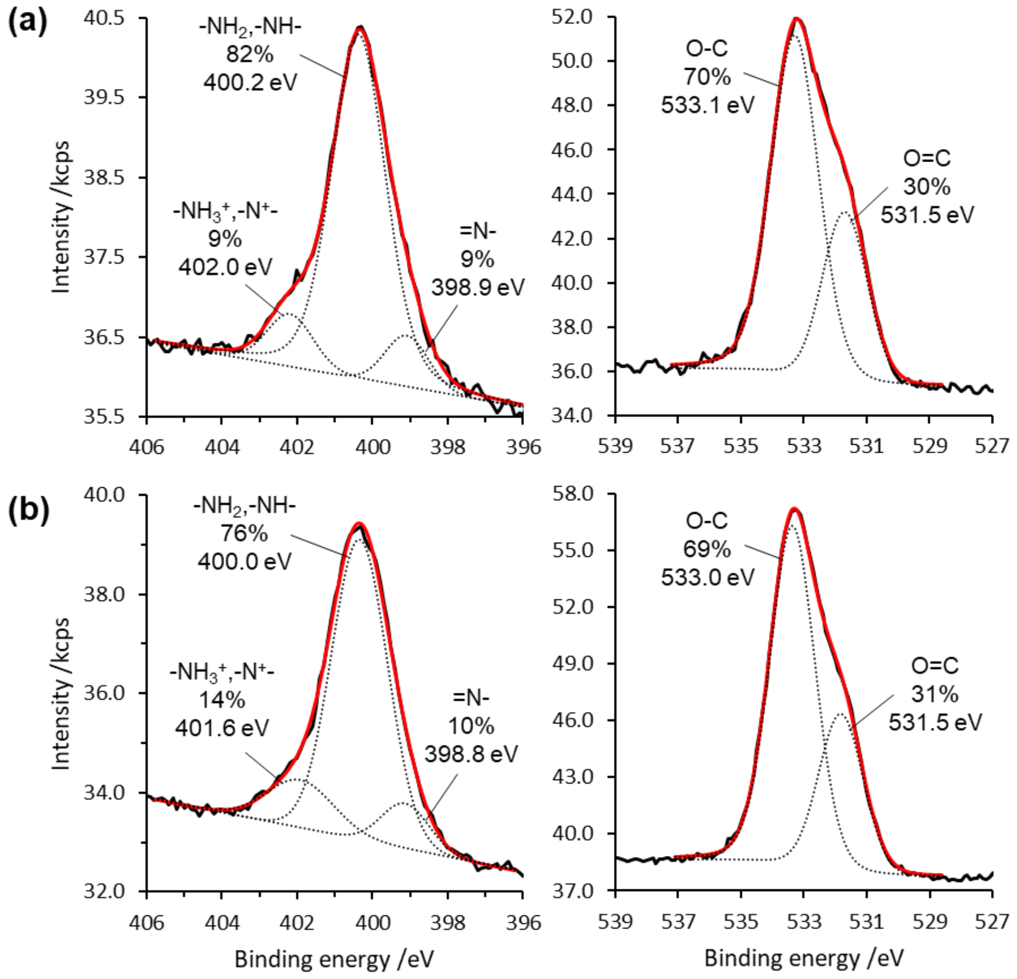

Fig. S3: XPS spectra of N 1s and O 1s regions of Au/ePDA **(a)** and Au/ePDA-ETA **(b)** modified electrodes. The percentages of each functional group and their peak binding energies are included in the figure as labels. In the N 1s spectrum three components are fitted, assigned to aromatic amines (=N-), aliphatic amines(-NH_2, -NH-) and to ammonium state (-NH_3^+, -N^+-), whereas in the O 1s spectrum two components are fitted assigned to carbonyl group (O=C) and to O-C simple bond [1-3].

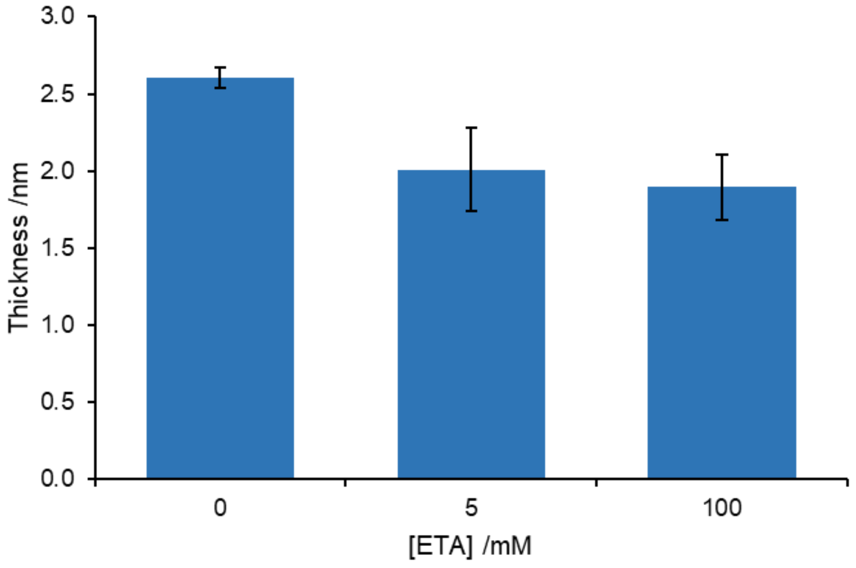

Fig. S4: Optical thickness of IgG layer deposited on gold modified with pristine polydopamine film (ePDA) and incubated polydopamine films in 5 and 100 mM of ethanolamine (ePDA/ETA).

References:

1. Almeida, L. C. *et al.* Electrosynthesis of polydopamine films - tailored matrices for laccase-based biosensors. *Applied Surface Science* **480**, 979–989 (2019).
2. Rella, S. *et al.* Investigation of polydopamine coatings by X-ray Photoelectron Spectroscopy as an effective tool for improving biomolecule conjugation. *Applied Surface Science* **447**, 31–39 (2018).
3. Zangmeister, R. A., Morris, T. A. & Tarlov, M. J. Characterization of polydopamine thin films deposited at short times by autoxidation of dopamine. *Langmuir* **29**, 8619–8628 (2013).
